# Religious affiliation and suicidality among college students in China: A cross-sectional study across six provinces

**DOI:** 10.1371/journal.pone.0251698

**Published:** 2021-05-19

**Authors:** Bob Lew, Kairi Kõlves, Jie Zhang, Wang Zhizhong, Harold G. Koenig, Paul S. F. Yip, Mansor Abu Talib, Augustine Osman, Ching Sin Siau, Caryn Mei Hsien Chan

**Affiliations:** 1 Australian Institute for Suicide Research and Prevention, Griffith University, Queensland, Australia; 2 Australian Institute for Suicide Research and Prevention, Queensland, Australia; 3 WHO Collaborating Centre for Research and Training in Suicide Prevention, Stockholm, Sweden; 4 School of Applied Psychology, Griffith University, Queensland, Australia; 5 Shandong University Centre for Suicide Prevention Research, Jinan, Shandong, China; 6 Department of Sociology, State University of New York College at Buffalo, Buffalo, New York, United States of America; 7 Department of Epidemiology and Statistics, School of Public Health at Guangdong Medical University, Dongguan, Guangdong, China; 8 Duke University Medical Center, Durham, North Carolina, United States of America; 9 King Abdulaziz University, Jeddah, Saudi Arabia; 10 Department of Social Work and Social Administration, The University of Hong Kong, Hong Kong, People’s Republic of China; 11 HKJC Centre for Suicide Research and Prevention, The University of Hong Kong, Hong Kong, People’s Republic of China; 12 Department of Human Development and Family Studies, Putra University of Malaysia, Serdang, Selangor, Malaysia; 13 Department of Psychology, University of Texas at San Antonio, San Antonio, Texas, United States of America; 14 Center for Community Health Studies, Faculty of Health Sciences, Universiti Kebangsaan Malaysia, Kuala Lumpur, Malaysia; University of New South Wales, AUSTRALIA

## Abstract

**Background:**

Several past studies indicated that religious beliefs, orientation, and practice are protective of suicide. Findings from recent studies in China suggest that religiosity may contribute to increased suicidality. However, few studies have examined the associations between religious affiliation across different faiths and suicidality in China.

**Objective:**

The current study examines the association between religious affiliation and suicidality among college students in six provinces in China.

**Methods:**

We conducted a cross-sectional study involving 11,407 college students from six universities in Ningxia, Shandong, Shanghai, Jilin, Qinghai, and Shaanxi. We collected the data between October 2017 and March 2018 using self-report questionnaires. They included self-report measures of depression, psychache, hopelessness, self-esteem, social support, and life purpose.

**Results:**

Participants with a Christian affiliation had 1.5 times (95% CI: 1.14, 1.99, *p* = 0.004) higher odds of indicating an elevated suicide risk, 3.1 times (95% CI: 1.90, 5.04, p<0.001) higher odds of indicating a previous suicide attempt, and increased overall suicidality (*B* = 0.105, *p* < 0.001) after accounting for demographic and risk/protective factors. Christians also scored the highest in depression, psychache, hopelessness, and the lowest social support, self-esteem, and purpose in life. Muslims reported decreased suicidality (*B* = -0.034, *p* = 0.031). Buddhism/Daoism yielded non-significant results in the multivariate analyses.

**Conclusions:**

Christian college students reported increased suicidality levels, perhaps due to public policies on religion. The decreased suicidality levels among Muslims may be attributed to higher perceived social support. The associations between religious affiliation and suicidality, depression, and hopelessness contrast sharply with US samples. This finding may be influenced by interactions between the religious denomination, individual, and social/political factors. This conclusion includes the possibility of anti-religious discrimination, which this paper did not investigate as a possible mediator and therefore remains a conjecture worthy of future investigation.

## Introduction

Suicide is a leading cause of death in young people in the 15- to 29-years age bracket worldwide [1]. A recent meta-analysis of 634,662 students by Mortier et al. [2] found that the pooled prevalence of lifetime suicidal ideation and suicide attempts were 22.3% and 3.2%, respectively. In China, a study by Yang and colleagues [3] indicated that the pooled prevalence of lifetime suicide attempts among 88,225 college students was 2.8%, with the highest rates recorded among rural students (5.1%). The risk of lifetime suicidal ideation and suicide attempts among college students was higher than adults’ prevalence rates worldwide [4].

Religion and religiosity have been proposed to be protective factors for suicide since Emile Durkheim’s study, which revealed that Protestants reported higher suicide rates than Catholics in late 20^th^ century Western Europe [5]. The connection between religion and emotional well-being has spurred numerous empirical studies over the past 50 years to understand further the complex relationships between religion and suicidality [6]. Religious affiliation, commitment, and practice are among the few independent factors reported to contribute substantially to lower the likelihood of individual suicidality [7, 8]. Religion has also been related to the country and regional suicide rates [9, 10], with most studies focusing on Western countries. In a cross-national study of 22 European countries, the results suggested that religion is a protective factor in the relatively secularized European nations and regions after controlling for economic level, economic strain, and education [11]. A study on 124 countries using the WHO data on suicide rates indicated that Islamic countries had significantly lower suicide rates than non-Islamic countries [12]. A meta-analysis on religion and completed suicide also showed that religion serves as a protective factor in Western countries but loses its protectiveness in Eastern countries [13].

A number of studies on religious affiliation and suicide among university students have revealed inconsistent findings. For example, a study in Australia found that a greater proportion of students affiliated to Protestantism and Catholicism reported experiencing suicidal ideation compared to the group with no religion. However, the no-religion group had a larger proportion of individuals who had attempted suicide [14]. Among Ukrainian college students, those with a religious affiliation had a lower likelihood of reporting lifetime suicide attempts compared to individuals with no religion [15]. In terms of inter-religion differences, a large-scale study on LGBQ university students found that Jewish-affiliated participants were less likely to report lifetime suicidal ideation, but non-religious participants were more likely to wish they were dead compared to Christian LGBQ participants [16]. Eskin et al.’s [17] study of university students across 12 countries showed that Muslims had a higher risk of attempting suicide while Buddhists, Catholics, and individuals with no religion had lower risks. These findings indicate that the association between religious affiliation and suicidality is an important area for further investigation, especially in non-Western settings such as China.

In China, only an estimated 15% of the population report a religious affiliation [18]. Studies examining associations between religion and suicide have gained momentum, although existing studies indicated mixed findings. For example, the age-standardized rate of lifetime suicidal ideation and lifetime suicide planning was significantly higher in the mostly Muslim Hui ethnic group than in the mainly atheist Han ethnic group [19]. Similarly, Zhang et al. [20] found that religious believers reported higher depression and suicidal ideation, suggesting that religion may be a risk factor for suicide. Lew et al. [21] and He et al. [22] found that religion may be a protective factor in their study of college students in Shandong and Northwestern China. Wang et al. [23], however, found no significant correlations between religious involvement and suicidal ideation, plan, and/or attempt in either Muslims or those not following any religion.

We have chosen to measure the type of religious affiliation as a risk/protective factor for suicidal thoughts and behaviors due to a few reasons. First, doctrinal beliefs regarding suicide vary between different religious beliefs and may influence suicidal behavior. For example, devotees of Christianity and Islam believe that suicide is a sin, and therefore a doctrinal commitment to these religions may serve as a suicide deterrent [24]. Compared to other religions, Muslims have the lowest level of permissiveness towards suicide [25]. In contrast, it has been found that Hindus and Buddhists hold more permissive views of suicide [26]. As past studies have indicated a positive relationship between attitudes toward suicide and suicidality, an individual who is affiliated with Islam may have different levels of suicidality compared to an individual who is affiliated with Hinduism or Buddhism. Secondly, the association between religious affiliation and suicidality needs to be examined against the background of the predominant culture of a locality. Eskin et al. [17] proposed that religiosity may lose its protectiveness in a culture where religious affiliation is compulsory or is normative. On the other hand, according to Hu et al. [27], the protectiveness of a religion against psychological distress depends on the social status of the religion. In their study, Christians were found to exhibit worse depression symptoms compared to Buddhists and individuals with no religion. This led them to hypothesize that self-identification with a marginalized religion may be a psychological burden [27]. Therefore, we aim to examine whether different religious affiliations among college students in China are associated with overall suicidal behavior, suicide attempt, and suicide risk.

For this study, religious affiliation is defined as the self-reported identification as belonging to a specific religious denomination (such as Buddhism/Daoism, Christianity, Islam, and other religions).

## Materials and methods

### Study design

We conducted a large cross-sectional cluster sampled study of undergraduate college students in six provinces in China.

### Data collection

Data were collected in a paper-based survey format from students enrolled in various undergraduate degrees from six provinces between October 2017 and March 2018: Ningxia, Shandong, Shanghai, Jilin, Qinghai, and Shaanxi. Ethics approval was obtained from the School of Public Health ethics committee, Shandong University (No. 20161103). One university was selected in each province through convenience sampling. Students from each department were clustered according to the year of study. An equal number of classes from each year of study were then selected to obtain a reasonable representation of each grade. All students in the selected classes were briefed about the research. To be eligible for participation in this study, an individual had to be at least 18 years of age and enrolled as a student from one of the six participating undergraduate programs. Participants completed the questionnaire anonymously in about half an hour, and no personal identifiers were collected. Participants were given a small gift equivalent to $1 in value upon completion of the questionnaire. All participants provided written informed consent. Hotlines on counseling services were provided in the information sheet tailored to each province.

### Questionnaire

#### Demographics

The following information was collected: age, gender, year of study, and religious denomination (Buddhism/ Daoism, Christianity, Islam, other religions, and not following a religion). No free text response field was provided to describe what ‘other’ religion participants were.

#### Suicidal Behaviors Questionnaire-Revised (SBQ-R)

The SBQ-R was developed as a brief measure of a range of suicide-related behaviors for use in both clinical and non-clinical settings. It is a 4-item self-report questionnaire [28]. The total score ranges from 3 to 18, with higher scores indicating a greater risk of suicidal thoughts and behaviors. This study uses a cut-off score of ≥ 7 to indicate suicide risk, based on the recommended cut-off score for undergraduate students by Osman et al.’s [28] validation study of the SBQ-R. As in studies in clinical and non-clinical settings, we used the SBQ-R Item 1 to determine lifetime suicide attempts: “Have you ever thought about or attempted to kill yourself?”. Participants who chose response options, “I have attempted to kill myself, but did not want to die” or “I have attempted to kill myself, and really hoped to die,” were identified as the lifetime suicide attempt participants. The Chinese translated version yielded internal consistency reliability of Cronbach’s α = 0.67 for the SBQ-R total scale score [29]. In this study, the Cronbach’s α of the scale score was 0.75 for the study samples.

#### Beck Hopelessness Scale

The Beck Hopelessness Scale was developed by Beck and colleagues [30] to measure three dimensions of the hopelessness construct: feelings about the future, motivation loss, and expectations. It is a 20-item questionnaire, ranging from 1 = “in full compliance with” to 5 = “completely opposite of” the stated item. The total scale score is usually derived to evaluate levels of the hopelessness construct; higher total scores represent higher levels of hopelessness. The estimate of internal consistency reliability is high (Cronbach’s α = 0.93) in a population of suicide attempters [30]. Scores derived from the scale are positively correlated with scores on measures of suicidality [31]. Yuan et al.’s [32] study among Chinese university students revealed the internal consistency reliability of Cronbach’s α = 0.90. In this study, the Cronbach’s α of the scale score was 0.80 for the study samples.

#### Psychache Scale

The Psychache Scale [33] consists of 13 items reflecting psychache (i.e., mental pain) scored from 1 = “never or strongly disagree” to 5 = “always or strongly agree.” The Psychache Scale scores have shown acceptable psychometric properties, with alpha reliability coefficients of 0.90 and higher when completed by university students [33]. In a sample of Chinese students, Cronbach’s α was 0.96 [34]. In this study, the Cronbach’s α of the scale score was 0.96 for the study samples.

#### DASS-21 depression subscale

DASS-21 is a well-established instrument comprising three psychological distress dimensions, including depression, anxiety, and stress [35]. Each dimension is composed of seven items. The score on each item ranges from 0 = “did not apply to me at all” to 3 = “applied to me very much,” or “most of the time”). The total scale score ranges from 0 to 63. Sub-scale scores can also be used to measure each dimension, which ranges from 0 to 21. The DASS-21 has been widely used in China for various psychosocial studies, such as Cheng et al. [36], which reported internal consistency reliability of Cronbach’s α = 0.77. For this study, we used the depression (DASS-Depression) sub-scale score in the analysis. The Cronbach’s α of the sub-scale score was 0.88 for the study samples.

#### Self-esteem scale

The self-esteem scale (SES), developed by Rosenberg [37], was originally used to assess adolescents’ overall feelings of self-worth and self-acceptance. The SES is currently the most widely used self-esteem measure of this construct and has an acceptable estimate of internal consistency reliability (Cronbach’s α = 0.84). Although commonly used with adolescents, the scale has also been used in a Chinese college student sample [38]. The SES consists of 10 items, with scaling ranging from 1 = “strongly disagree” to 4 = “strongly agree.” The total scale score ranges from 10 to 40. Higher scores indicate more significant levels of self-esteem. The Chinese SES was tested in China and had an acceptable estimate of internal consistency reliability (Cronbach’s α = 0.84) [38]. In this study, the Cronbach’s α of the scale score is 0.65 for the study samples.

#### Social support

The 12-item Multidimensional Scale of Perceived Social Support (MSPSS) was used to measure social support. Each item is scored using a 7-point Likert-type scale ranging from 1 = “very strongly disagree” to 7 = “very strongly agree,” resulting in a total score range from 12 to 84 [39]. The scale has acceptable reliability and validity estimates in the Chinese context [40, 41]. The MSPSS measures social support from three social support sources, including family members, friends, or significant others (or a special person). Higher scores on this scale suggest a higher level of social support [39]. The total score is used to assess the overall total support from the three identified sources. In this study, the Cronbach’s α of the scale score was 0.94 for the study samples.

#### Purpose in life

The four-item purpose in life test–short form (PLT-SF) was used to measure the extent to which participants felt their lives had meaning and purpose [42]. The PLT-SF includes a 7-point Likert-type scale response format. Responses to the items are summed to obtain a total scale score ranging from 4 to 28. Higher scores suggest greater perceived meaning/purpose in life. A Chinese version of the PLT-SF has been shown to have an acceptable internal consistency reliability estimate (Cronbach’s α = 0.89) [43]. In this study, the Cronbach’s α of the scale score was 0.92 for the study samples.

### Statistical analysis

Univariate and multivariate statistical analyses were conducted using the IBM SPSS v.21 (SPSS Inc.; Armonk, NY). The participant demographics were computed using descriptive statistics. Chi-square tests were used to examine the associations between type of religion and suicide risk and suicide attempt. We used a series of one-way analyses of variance (ANOVAs) to examine mean group differences between participants who reported “not following a religion” (reference group) and those who identified a specific religious affiliation on the study self-report measures. These were scores for risk (hopelessness, psychache, depression) and protective (self-esteem, social support, and purpose in life) measures.

We extended the analyses by conducting a multiple regression analysis to assess the impact of religious affiliation on suicidality, adjusting for demographic factors (age and gender), province, and psychological distress factors as covariates. Specifically, the SBQ-R total score was used in the analysis to measure suicidal thoughts and behavior. All potential continuous predictors were mean-centered (except age) before they were included in the analyses.

A multiple logistic regression analysis was conducted to measure the odds of being at risk for suicide (SBQ-R total score ≥ 7) for participants from each religious affiliation. Using the recommended total SBQ-R cut-off score of 7 or higher, 1,801 (18.8%) participants met the criteria for suicide risk (coded = 1); those with scores below this cut-off were assigned to the control or reference group (n = 7,801, coded as 0). These analyses included all the study participants (N = 9,602). Participants’ age, gender, province, and scores on the risk and protective self-report measures were covariates in the analysis.

Finally, unlike the previous analyses, the subsequent analysis used scores on the SBQ-R Item 1 (“Have you ever thought about or attempted to kill yourself?”). A total of 216 (2.2%) participants reported a lifetime suicide attempt (coded = 1); those who did not report a lifetime suicide attempt were assigned to the control or reference group (n = 9,386, coded as 0). These analyses included all the study participants (N = 9,602). A multiple logistic regression analysis was conducted to measure the odds of a lifetime of suicide attempt (based on their response to the SBQ-R Item 1, indicating a lifetime suicide attempt) for participants from each religious affiliation. Participants’ age, gender, province, and scores on the risk and protective self-report measures were covariates in the analysis.

For all comparisons, differences were determined using two-tailed tests, while p-values less than 0.05 were considered statistically significant. Missing data were deleted list-wise during the statistical analysis.

## Results

### Demographics

A total of 11,407 college students from universities in the six Chinese provinces of Ningxia, Shandong, Shanghai, Jilin, Qinghai, and Shaanxi participated in this study. Cases with missing data, including gender, age, or any item of the SBQ-R instruments and religious denomination, were not included. In total, 9,602 responses were analyzed (mean age = 20.66±1.33; 59.8% female). Of these, there were 33.0%, 33.5%, and 31.4% participants in their first, second, and third years of college or above, respectively. Most participants reported no affiliation with any particular religion (71.3%). The type of religion varied significantly between provinces (χ^2^_(20)_ = 3348.09, p<0.001), with Shandong participants reporting the highest percentage of having no religion (88.0%) compared to the lowest in Qinghai (48.6%). Ningxia had the highest number of Muslims (40.4%), Shaanxi the highest number of Christians (8.2%), and Qinghai the highest number of Buddhists/Daoists (35.3%). In terms of gender breakdown by religion, 29.6% out of 5,746 females and 27.7% out of 3,856 males reported a religious affiliation.

### Suicidality by religion

Suicidality (i.e., suicidal thoughts and behaviors) which was measured using the SBQ-R total score, was the highest in the Shanghai province (5.38±2.80) and lowest in the Shandong province (4.32±2.28). Similarly, the percentage of participants reporting suicide risk was the highest in Shanghai province (28.1%) and the lowest in Shandong province (14.9%), χ^2^_(5)_ = 121.41, p<0.001 (Refer Table 1). Christians reported significantly higher suicidality score (5.71±3.30) compared to those affiliated with Buddhism/Daoism (4.87±2.68), other religions (4.72±2.61), individuals reporting not following a religion (4.67±2.44), and Islam (4.43±2.39). At the same time, Muslims had lower levels of suicidality than Buddhists/Daoists and those with no religion, *F*_(4, 9601)_ = 17.82, p < 0.001, η^2^ = 0.007. Similarly, a higher percentage of Christians were at risk of suicide (30.4%; χ^2^_(4)_ = 39.34, p < .001) and had attempted suicide (7.5%; χ^2^_(4)_ = 48.95, p<0.001) (Table 1).

**Table 1:** Suicidality measures by province and religious denomination.

Province	Religious denomination	*n* (%)	SBQ-R (Mean±SD)	SBQ-R At Risk (%)	Suicide Attempt (%)
Ningxia	Buddhism or Daoism	156 (8.5%)	4.63±2.15	18.6	1.9
	Christianity	17 (0.9%)	4.18±1.81	5.9	0.0
	Islam	737 (40.4%)	4.31±2.21	14.1	1.5
	Others	3 (0.2%)	4.92±2.91	25.0	0.0
	With religious affiliation (Sub-Total)	913 (50.5%)			
	Not following a religion	904 (49.5%)	4.59±2.42	17.5	2.7
	Total	1826 (100.0%)	4.48±2.32	16.2	2.1
Shandong	Buddhism or Daoism	55 (5.0%)	4.51±2.49	12.7	1.8
	Christianity	23 (2.1%)	4.74±2.28	21.7	0.0
	Islam	28 (2.5%)	4.96±2.87	25.0	0.0
	Others	26 (2.4%)	4.96±2.99	23.1	7.7
	With religious affiliation (Sub-Total)	132 (12.0%)			
	Not following a religion	972 (88.0%)	4.27±2.22	14.4	1.5
	Total	1104 (100.0%)	4.32±2.28	14.9	1.7
Shanghai	Buddhism or Daoism	141 (8.6%)	6.15±3.19	38.3	5.0
	Christianity	54 (3.3%)	6.76±3.61	44.4	7.4
	Islam	24 (1.5%)	6.63±4.19	37.5	4.2
	Others	29 (1.8%)	6.10±3.31	44.8	3.4
	With religious affiliation (Sub-Total)	248 (15.2%)			
	Not following a religion	1383 (84.8%)	5.22±2.65	26.0	1.2
	Total	1631 (100.0%)	5.38±2.80	28.1	1.8
Jilin	Buddhism or Daoism	141 (7.6%)	5.33±2.89	22.0	4.3
	Christianity	56 (3.0%)	4.96±2.38	21.4	3.6
	Islam	19 (1.0%)	4.47±2.37	10.5	5.3
	Others	7 (0.4%)	4.57±2.44	14.3	0.0
	With religious affiliation (Sub-Total)	223 (12.0%)			
	Not following a religion	1640 (88.0%)	4.62±2.16	15.9	1.4
	Total	1863 (100.0%)	4.68±2.24	16.5	1.7
Qinghai	Buddhism or Daoism	495 (35.3%)	4.50±2.41	17.8	1.6
	Christianity	27 (4.0%)	4.81±2.34	37.0	7.4
	Islam	151 (10.8%)	4.79±2.63	27.8	6.0
	Others	48 (3.4%)	4.10±1.95	8.3	2.1
	With religious affiliation (Sub-Total)	721 (51.4%)			
	Not following a religion	682 (48.6%)	4.64±2,62	23.0	2.3
	Total	1403 (100.0%)	4.59±2.52	22.2	2.6
Shaanxi	Buddhism or Daoism	311 (17.5%)	4.86±2.84	19.6	4.5
	Christianity	145 (8.2%)	6.11±3.72	33.8	11.0
	Islam	38 (2.1%)	3.58±1.97	7.9	2.6
	Others	17 (1.0%)	3.65±0.79	0.0	0.0
	With religious affiliation (Sub-Total)	511 (28.8%)			
	Not following a religion	1264 (71.2%)	4.50±2.50	15.5	2.5
	Total	1775 (100.0%)	4.67±2.70	17.4	3.5
Total	Buddhism or Daoism	1299 (13.5%)	4.87±2.68	20.8	3.0
	Christianity	322 (3.4%)	5.71±3.30	30.4	7.5
	Islam	997 (10.4%)	4.43±2.39	15.8	2.4
	Others	139 (1.4%)	4.72±2.61	20.1	2.9
	Not following a religion	6845 (71.3%)	4.67±2.44	18.2	1.8
	Total	9602 (100.0%)	4.71±2.51	18.8	2.2

^Notes. *n* = frequency. *SD* = standard deviation. SBQ-R = Suicidal Behaviors Questionnaire-Revised. Suicide risk is based on a total score of 7 and above on the SBQ-R.^

### Religion and risk/protective factors for suicidality

Results of the one-way ANOVAs indicated that risk factors for suicide such as the level of hopelessness were significantly higher among all religions compared to those with no religion, except for Islam (*F*_(4, 9446)_ = 28.45, *p*<0.001, η^2^ = 0.011). Psychache (*F*_(4, 9460)_ = 29.05, *p*<0.001, η^2^ = 0.012) and depression (*F*_(4, 9517)_ = 29.34, *p*<0.001, η^2^ = 0.012) were significantly higher for Christians, Buddhists/Daoists, and those with other religions. Compared to all other religions in this study, Christians and Buddhists/Daoists reported lower protective factors against suicidality, specifically in terms of self-esteem (*F*_(4, 9479)_ = 16.09, *p*<0.001, η^2^ = 0.007), social support (*F*_(4, 9482)_ = 20.64, *p*<0.001, η^2^ = 0.009), and purpose in life (*F*_(4, 9542)_ = 33.67, *p*<0.001, η^2^ = 0.014) (Table 2).

**Table 2:** One-way ANOVA analysis comparing hopelessness, psychache, depression, self-esteem, social support, and purpose in life by religious denomination.

Religious Denomination	Hopelessness	Psychache	Depression	Self Esteem	Social Support	Purpose in Life
Buddhism/Daoism	52.53±9.06***	27.08±10.57***	5.85±4.45***	26.34±3.43***	60.39±13.45***	19.44±5.20***
Christianity	54.37±8.92***	28.13±10.22***	6.60±4.77***	26.08±3.06***	56.86±14.82***	17.72±5.76***
Islam	49.50±9.66**	24.02±9.72	4.66±4.02	27.14±3.49	62.82±11.46	20.43±4.51
Other religions	53.59±9.46**	26.69±10.15*	6.31±5.06***	26.52±3.75	61.26±12.10	19.70±5.28
Not following a religion	50.73±9.54	24.43±11.44	4.87±4.28	27.07±3.47	62.25±12.18	20.34±4.44
Total	51.01±9.53	24.90±10.25	5.05±4.33	26.94±3.67	61.87±12.44	20.13±4.65

^Notes.^

* ^*p* < .05;^

** ^*p* < .01;^

*** ^*p* < .001.^

^Depression is measured using DASS-21 Depression sub-scale. Dunnett post-hoc analyses were conducted using “Not following a religion” as the reference group.^

The results of the multiple linear regression suggested that, after adjusting for age, gender, province, hopelessness, depression, psychache, self-esteem, social support, and purpose in life, those with a Christian religious affiliation reported higher suicidality (suicidal thoughts and behavior) levels (*B* = 0.105, *p* < 0.001), whereas Muslims had lower suicidality levels (*B* = -0.034, *p* = 0.031). Together, the independent variables accounted for 19.2% of the variance, *R*^2^ = 0.192, adjusted *R*^2^ = 0.190, *F*_(17, 9046)_ = 126.43, *p*<0.001 (Table 3).

**Table 3:** Multiple linear regression analysis for factors predicting suicidality among college students.

Variable	*B*	*B SE*	95% Confidence Interval		*β*	*t*	*p*-value
			Lower	Upper			
**Constant**	0.196	0.068				2.866	0.004
**Religious Denomination**							
Buddhism/Daoism	0.017	0.013	-0.008	0.042	0.014	1.357	0.175
Christianity	0.105	0.023	0.060	0.149	0.045	4.623	<0.001
Islam	-0.034	0.016	-0.065	-0.003	-0.024	-2.158	0.031
Other religions	0.024	0.036	-0.046	0.094	0.006	0.663	0.507
Not following a religion ^†^							
**Age**	-0.015	0.003	-0.021	-0.008	-0.045	-4.574	<0.001
**Gender**							
Female	0.073	0.009	0.056	0.090	0.083	8.512	<0.001
Male^†^							
**Province**							
Ningxia	0.083	0.015	0.054	0.112	0.075	5.589	<0.001
Shandong	0.024	0.016	-0.007	0.056	0.018	1.522	0.128
Shanghai	0.050	0.005	0.041	0.059	0.133	10.824	<0.001
Jilin	0.103	0.013	0.077	0.130	0.097	7.649	<0.001
Qinghai	-0.001	0.015	-0.030	0.029	-0.001	-0.048	0.961
Shaanxi ^†^							
Hopelessness	-0.002	0.001	-0.003	0.000	-0.037	-2.784	0.005
Psychache	0.009	0.001	0.008	0.010	0.207	14.672	<0.001
Depression	0.015	0.001	0.012	0.018	0.153	10.193	<0.001
Self-esteem	-0.009	0.001	-0.012	-0.007	-0.080	-6.593	<0.001
Social Support	0.001	0.000	0.000	0.002	0.027	2.419	0.016
Purpose in Life	-0.011	0.001	-0.013	-0.009	-0.121	-10.323	<0.001

^Notes. *F* (17, 9046) = 126.43, *p*<0.001. Suicidality was measured by the participant’s total score on the Suicidal Behaviors Questionnaire-Revised. *B* = unstandardized beta. *SE* = standard error. β = standardized beta.^

^†Reference group.^

A multiple logistic regression analysis was conducted with suicide risk as the dependent. The full model was statistically significant, (Chi-square _(17)_ = 1344.68, p<0.001). This model accounted for 22.2% of the variance in suicide risk (Nagelkerke R^2^ = 0.222). Together, the model correctly identified 81.7% of the participants with suicide risk. The results indicated that participants affiliated with a Christian denomination had 1.5 times higher odds of having an elevated suicide risk (OR: 1.51, 95% CI: 1.14, 1.99, *p* = 0.004) (Table 4).

**Table 4:** Multiple logistic regression analysis for factors predicting suicide risk (based on a total score of 7 and above on the Suicidal Behaviors Questionnaire-Revised) among college students.

Variable	Wald	Odds Ratio	95% Confidence Interval		*p*-value
			Lower	Upper	
**Constant**	1.889	0.504			
**Religious Denomination**					
Buddhism/Daoism	0.007	1.007	0.845	1.202	0.934
Christianity	8.312	1.505	1.140	1.986	0.004
Islam	2.377	0.834	0.661	1.051	0.123
Other religions	0.272	1.136	0.703	1.835	0.602
Not following a religion ^†^					
**Age**	12.849	0.920	0.879	0.963	<0.001
**Gender**					
Female	60.736	1.647	1.452	1.867	<0.001
Male^†^					
**Province**					
Ningxia	25.674	1.754	1.411	2.180	<0.001
Shandong	8.155	1.420	1.116	1.805	0.004
Shanghai	65.610	1.292	1.215	1.375	<0.001
Jilin	11.262	1.403	1.151	1.709	0.001
Qinghai	4.796	1.269	1.025	1.571	0.029
Shaanxi ^†^					
Hopelessness	0.778	0.996	0.987	1.005	0.378
Psychache	142.108	1.048	1.040	1.056	<0.001
Depression	69.623	1.086	1.065	1.108	<0.001
Self-esteem	18.227	0.956	0.936	0.976	<0.001
Social Support	0.082	1.001	0.995	1.006	0.775
Purpose in Life	60.987	0.944	0.930	0.958	<0.001

^Note. Chi-square (17) = 1344.68, p<0.001. Nagelkerke *R* square = 0.222.^

^†Reference group.^

Another multiple logistic regression analysis was conducted with lifetime suicide attempts as the dependent. The full model was statistically significant, (Chi-square _(17)_ = 218.36, p<0.001). This model accounted for 12.6% of the variance in suicide attempts (Nagelkerke R^2^ = 0.126). Together, the model correctly identified 97.8% of the participants with suicide attempts. The results indicated that Christian participants had 3.1-times higher odds of reporting a previous suicide attempt (OR: 3.09, 95% CI: 1.90, 5.04, p<0.001; Table 5).

**Table 5:** Multiple logistic regression analysis for factors predicting suicide attempt among college students.

Variable	Wald	Odds Ratio	95% Confidence Interval		*p*-value
			Lower	Upper	
**Constant**	0.019	0.843			
**Religious Denomination**					
Buddhism/Daoism	2.145	1.355	0.902	2.034	0.143
Christianity	20.498	3.093	1.897	5.044	<0.001
Islam	0.501	1.227	0.696	2.164	0.479
Other religions	1.032	1.714	0.606	4.843	0.310
Not following a religion ^†^					
**Age**	9.900	0.829	0.738	0.932	0.002
**Gender**					
Female	0.167	1.065	0.786	1.443	0.683
Male^†^					
**Province**					
Ningxia	0.338	0.857	0.511	1.440	0.561
Shandong	2.155	0.641	0.353	1.161	0.142
Shanghai	10.287	0.771	0.658	0.904	0.001
Jilin	5.355	0.571	0.355	0.918	0.021
Qinghai	1.824	0.721	0.449	1.159	0.177
Shaanxi ^†^					
Hopelessness	3.648	0.978	0.956	1.001	0.056
Psychache	28.592	1.050	1.031	1.069	<0.001
Depression	20.135	1.109	1.060	1.161	<0.001
Self-esteem	0.219	1.012	0.962	1.066	0.640
Social Support	5.233	0.986	0.973	0.998	0.022
Purpose in Life	0.058	1.004	0.973	1.036	0.810

^Note. Chi-square (17) = 218.36, p<0.001. Nagelkerke *R* square = 0.126.^

^†Reference group.^

## Discussion

Our key finding suggests that religious denomination may be either a risk or a protective factor for suicidality. It should be noted, however, that suicide risk per se was low amongst this sample. Specifically, being of the Christian faith appeared to be associated with increased suicidality, suicide risk, and suicide attempt. These results are not consistent with past findings among college students in Western countries, where affiliation to Catholic and Protestant Christianity was protective against suicidality [14], and those who professed a religious affiliation were less likely to have attempted suicide [15]. Compared to other religions, being of Christian faith appeared to be associated with higher levels of hopelessness, psychache, and depression, as well as lower levels of self-esteem, social support, and purpose in life, all of which are known risk and protective factors for suicidality. The higher depression level among the Christian participants is comparable to the findings of a study by Hu et al. [27], where it was also found that Christians in China’s general population were more likely to be depressed compared to Buddhists and atheists. Other studies in China have also revealed that increased depression and hopelessness, and decreased social capital were predictive of increased suicidality [44, 45]. Taking these findings into consideration, the greater suicide risk among the Christian participants in our study could originate from their worse psychological health. This may be because adherence to the Christian faith places an individual in a minority group in China, as the Christian population in China is small and loosely spread across China [46]. For example, in this sample, most Christians were Han Chinese, which made them a minority (3.7%) compared to the majority of Han Chinese who did not profess any religious affiliation (83.0%). Also, Christians reported the lowest level of social support in this sample, indicating feelings of exclusion.

In contrast, being Muslim was found to be protective against suicidality, given the lowest scores in hopelessness. Unlike Christians, who are a minority within their nation and race, Muslims are a majority within the Hui race in this sample (85.8%). Even though Islam is not the majority religion in China, the concentration of Muslims in Northwestern China (e.g., Ningxia, Qinghai, and Gansu) is high [46].

The role of religion as a protective or risk factor for suicidality may be explained by Rodney Stark’s moral community hypothesis, which postulates that the aggregate level of religiousness in a group (e.g., neighborhood, community, city, country, state, region, and nation) will affect the attitudes and behaviours of its members, whether or not they are affiliated with a religion [47]. The moral community hypothesis found mixed support in a study on Chinese college students, where provinces that had a higher number of mosques (indicating a higher level of aggregate religiosity among Muslims) were associated with lower odds of deviant behaviour, but the relationship was not significant where Christianity and Buddhism were examined [46]. Similarly, in a community or province with higher religiousness, the likelihood of religion being a protective factor against suicidality will be higher. The finding also means a country or community with low religious rates, such as China may have lower religiosity, and this may lead to religion having a lower or no protective effect against suicidality. This hypothesis, however, remains tentative and requires further evaluation in the future.

However, given the low rate of religious affiliation in China, and also its repressive political and social policy towards religion [18], another alternative hypothesis we propose is the “religious minority hypothesis”. This hypothesis can characterize an environment where religious affiliation is low in the community, province, and nation, and the practice of religion is reported to be tightly controlled by the government. China, a communist country, has the largest population in the world, with 1.4 billion people, but it has the world’s lowest religion rate estimated to be about 15% [18]. The Chinese government has been reported to restrict the practice of religion and does not include religious elements into its public policy and decision-making processes [18]. Religious freedom is actively managed/controlled. In such an environment, it may be detrimental or even stressful to profess too strongly in a religious belief and/or to practice religion openly. This may be a root cause leading to a sense of discrimination, decreased social integration and connectivity, low belongingness to the society they interact with, disturbance in family harmony, social isolation, and clashes in value systems and aspirations. The restrictions imposed may have led to religion losing its protective effect against suicidality, and in our sample, Christians and Buddhists/Daoists even reported higher levels of suicidality and/or risk factors. Thus, the religious minority hypothesis may help to explain why certain religions are not protective against suicidality. Islamic adherents, however, are more likely to form close social support networks, offering a stronger sense of community and cohesion [47], which could serve as a protective factor, thus lowering suicide risk and negating other negative psychological factors. However, as stated in the moral community hypothesis, there is a potential for these suicidal risks to be stemming from regional differences, which is also tied to the religious demographics of the region. Therefore, this hypothesis should be subjected to further research.

The study has a number of limitations. First, with the cross-sectional design, inferences of causation cannot be drawn. Second, this study is limited to undergraduate students in urban provinces we sampled and is not generalizable to all universities in China. The religion rate reported in our study should not be assumed to represent the religion rate of any particular province or the general population in China. In addition, we did not measure the strength of the participants’ religious affiliation. A number of factors which has been shown to influence the association between religion and suicide were not included in our analysis, such as the acceptability of suicide [48]. We also did not examine Chinese values and philosophies such as Confucianism, which has been associated with suicide among young people in rural China [49]. Next, given the larger number of participants who reported not following a religion, the statistical methods employed may suffer from the problem of unequal variances.

Many previous studies of suicidality have been conducted in the US and Western European countries, as well as countries with a relatively higher rate of religious affiliation and a dominant religion. Future studies should involve other provinces in China, other age groups, and other relevant subgroups of the population to determine reasons why only Islam appears to be a protective factor, and whether this finding is due to social integration, specific beliefs, religious network, or moral community. Future studies could test our “religious minority hypothesis” in other countries with unique social and political atmospheres, and also to determine whether this pattern is generalizable throughout China. Finally, other aspects of religious affiliation which have been found to be related to suicidality, such as religious importance [50] could also be taken into account in future studies.

### Study implications

The association between religion and suicidality is dependent on interactions between the religious denomination, individual factors and the environment in which the individual is practicing the religion. Therefore, we should not generalize past studies which indicated that religion is protective of suicidality across national boundaries, especially for a country such as China with its varied sociodemographic, political and cultural characteristics [51]. That religion may serve as a risk or protective factor in different contexts is important to be noted in health policy-making on suicide prevention, clinical practice, and religious interventions for suicide. This study found that, within the university setting, there are associations between religious affiliation, regional variation, and suicidality. Therefore, religious and regional considerations should be incorporated into prevention and intervention efforts of student mental health care systems.

## Supporting information

S1 File (SAV)
